# Predictors of chronic COVID-19 symptoms in a community-based cohort of adults

**DOI:** 10.1371/journal.pone.0271310

**Published:** 2022-08-04

**Authors:** Jonathan I. Silverberg, Israel Zyskind, Hiam Naiditch, Jason Zimmerman, Aaron E. Glatt, Abraham Pinter, Elitza S. Theel, Michael J. Joyner, D. Ashley Hill, Miriam R. Lieberman, Elliot Bigajer, Daniel Stok, Elliot Frank, Avi Z. Rosenberg

**Affiliations:** 1 Department of Dermatology, George Washington University School of Medicine and Health Sciences, Washington, DC, United States of America; 2 Department of Pediatrics, NYU Langone Medical Center, New York, NY, United States of America; 3 Maimonides Medical Center, Brooklyn, NY, United States of America; 4 Department of Medicine, Yale University School of Medicine, New Haven, CT, United States of America; 5 Department of Medicine, Mount Sinai South Nassau and the Icahn School of Medicine at Mount Sinai, New York, NY, United States of America; 6 Public Health Research Institute, New Jersey Medical School, Rutgers, The State University of New Jersey, Newark, NJ, United States of America; 7 Division of Clinical Microbiology, Department of Laboratory Medicine and Pathology, Mayo Clinic, Rochester, MN, United States of America; 8 Department of Anesthesiology & Perioperative Medicine, Mayo Clinic, Rochester, MN, United States of America; 9 ResourcePath, Sterling, VA, United States of America; 10 Department of Dermatology, State University of New York Downstate Medical Center, New York, NY, United States of America; 11 Division of Gastroenterology, Department of Medicine, Brookdale University Hospital and Medical Center, Brooklyn, NY, United States of America; 12 Memorial Sloan Kettering Cancer Center, New York, NY, United States of America; 13 Division of Infectious Diseases, Department of Medicine, Jersey Shore University Medical Center, Neptune, NJ, United States of America; 14 The Hackensack Meridian School of Medicine, Clifton, New Jersey, United States of America; 15 Department of Pathology, Johns Hopkins University, Baltimore, MD, United States of America; British Columbia Centre for Excellence in HIV/AIDS, CANADA

## Abstract

**Background:**

COVID-19 can cause some individuals to experience chronic symptoms. Rates and predictors of chronic COVID-19 symptoms are not fully elucidated.

**Objective:**

To examine occurrence and patterns of post-acute sequelae of SARS-CoV2 infection (PASC) symptomatology and their relationship with demographics, acute COVID-19 symptoms and anti-SARS-CoV-2 IgG antibody responses.

**Methods:**

A multi-stage observational study was performed of adults (≥18 years) from 5 US states. Participants completed two rounds of electronic surveys (May-July 2020; April-May 2021) and underwent testing to anti-SARS-CoV-2 nucleocapsid protein IgG antibody testing. Latent Class Analysis was used to identify clusters of chronic COVID-19 symptoms.

**Results:**

Overall, 390 adults (median [25%ile, 75%ile] age: 42 [31, 54] years) with positive SARS-CoV-2 antibodies completed the follow-up survey; 92 (24.7%) had ≥1 chronic COVID-19 symptom, with 11-month median duration of persistent symptoms (range: 1–12 months). The most common chronic COVID-19 symptoms were fatigue (11.3%), change in smell (9.5%) or taste (5.6%), muscle or joint aches (5.4%) and weakness (4.6%). There were significantly higher proportions of ≥1 persistent COVID-19 symptom (31.5% vs. 18.6%; Chi-square, P = 0.004), and particularly fatigue (15.8% vs. 7.3%, P = 0.008) and headaches (5.4% vs. 1.0%, P = 0.011) in females compared to males. Chronic COVID-19 symptoms were also increased in individuals with ≥6 acute COVID-19 symptoms, Latent class analysis revealed 4 classes of symptoms. Latent class-1 (change of smell and taste) was associated with lower anti-SARS-CoV-2 antibody levels; class-2 and 3 (multiple chronic symptoms) were associated with higher anti-SARS-CoV-2 antibody levels and more severe acute COVID-19 infection.

**Limitations:**

Ambulatory cohort with less severe acute disease.

**Conclusion:**

Individuals with SARS-CoV-2 infection commonly experience chronic symptoms, most commonly fatigue, changes in smell or taste and muscle/joint aches. Female sex, severity of acute COVID-19 infection, and higher anti-SARS-CoV-2 IgG levels were associated with the highest risk of having chronic COVID-19 symptoms.

## Introduction

Acute COVID-19 disease, caused by the SARS-CoV-2 infection, is associated with heterogeneous clinical manifestations. While most individuals with COVID-19 develop acute symptoms that are self-limited, some will experience chronic COVID-19 symptoms well beyond the initial period of acute infection [1, 2]. Chronic (post-acute sequelae of SARS-CoV2 infection [PASC] or “long-haul”) COVID-19 symptoms can affect as many as one half of all patients, including fatigue, muscle weakness, loss of taste and smell, headaches, needle pains in arms and legs, diarrhea and bouts of vomiting, cough, and even shortness of breath and chest pain [1–4]. PASC is likely to represent a substantial public health burden in the coming months to years [5].

Several studies have sought to characterize the symptoms of chronic COVID-19 symptoms, their duration and the likelihood of occurrence after acute COVID-19 [1, 2, 4]. However, few studies, if any, described chronic COVID-19 as a syndrome comprised of specific symptom clusters. Even fewer identified associations of such symptom clusters with serologic evidence of past COVID-19 infection. Moreover, it remains to be seen which features of acute COVID-19 infection might predict the occurrence of any chronic COVID-19 symptoms in general or specific symptoms [3]. This study sought to gain insight into these questions in a large community-based cohort.

## Materials and methods

### Study design

This study involved a three-stage sampling design as previously described [6]. The baseline study was designed to determine the self-reported symptoms, outcomes and seroprevalence of SARS-CoV-2 in an ambulatory cohort of adults. The Multi-Institutional Study Analyzing anti-CoV-2 Antibodies (MITZVA) cohort recruited study participants in partnership with local non-for-profit and social service organizations offering antibody testing to symptomatic or asymptomatic adults within the large urban/suburban Orthodox Jewish communities of Brooklyn, NY, Lakewood, NJ, Los Angeles, CA, Nassau and Sullivan Counties, NY, New Haven, CT and Detroit, MI between May 13 and July 6, 2020 early in the pandemic and prior to the availability of COVID-19 vaccines. This particular ethno-religious group tends to live in concentrated geographic areas, is typically close-knit and holds religion to be a central part of their lives, ideally allowing for robust recruitment via religiously-affiliated venues [7]. Participants were recruited via paper and social media advertisement that were distributed by local non-for-profits and social service organizations with established networks of Orthodox Jewish community members. All members of these organizations self-identified with the Jewish community. Recruitment material provided a website address to enroll in this study. There was no compensation for participation.

**Fig 1:**
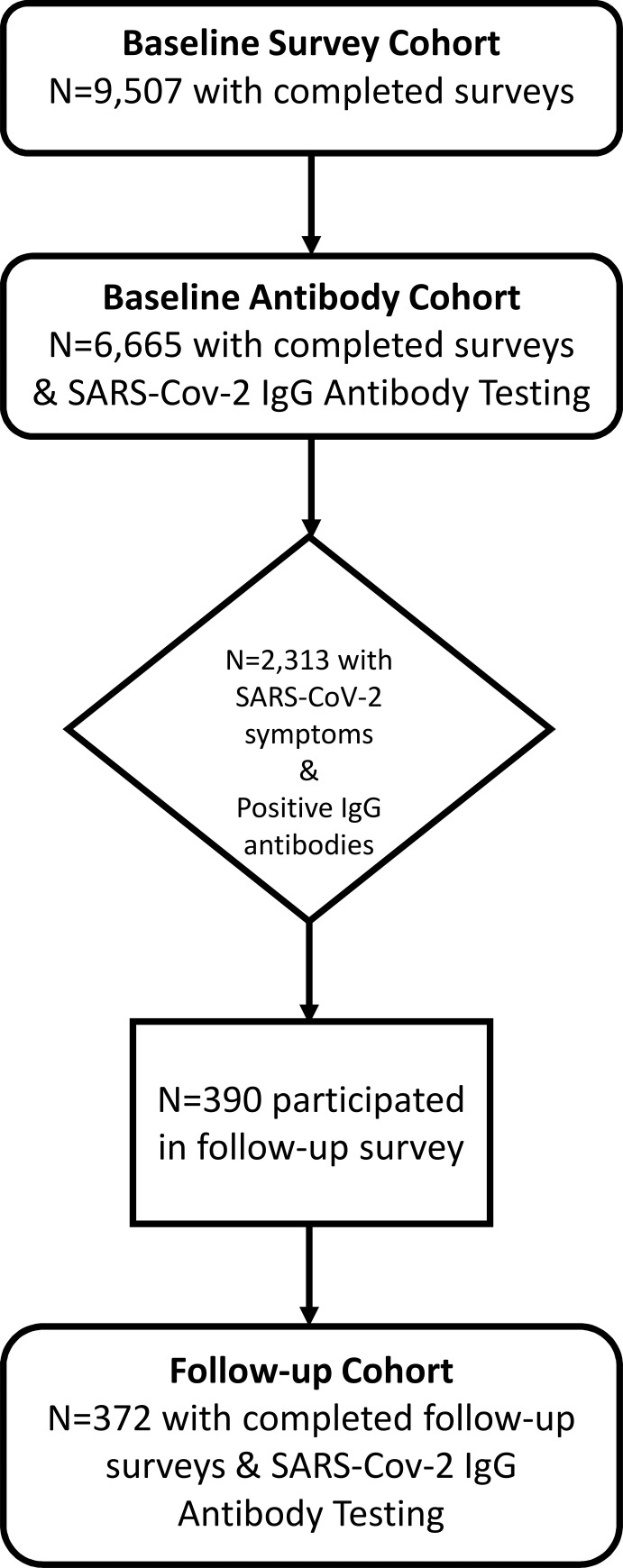
Cohort design.

Study inclusion criteria included age >18 years, male or female, ability to sign informed consent and for those participating in the synchronous antibody testing drive the release of antibody data to the study investigators. Exclusion criteria were those who were not able to complete the survey or did not agree to release their antibody data to the investigators. Participants, after inclusion and exclusion criteria were applied, were given a specific time and location to appear for serological testing. A cross-sectional survey invitation was sent to adults (age ≥18 years); 12,626 individuals began the survey process, with 9,507 adults completing the survey (completion rate = 75.4%)(Fig 1). Acute COVID-19 symptoms assessed included fever, cough, muscle ache, anosmia, dysgeusia, headache, diarrhea, vomiting, abdominal pain, and rash. In stage 2, a subset of 6,665 adults (70.1% response rate) had antibody testing performed shortly after completing the survey. Blood samples were collected at blood-drives in local community centers and transported in batches to Mayo Clinic for anti-SARS-CoV-2 testing. Electronic informed consent and disclosure of the study purpose was performed prior to beginning the survey. The study was open to all participants and did not require participants to have SARS-CoV-2 symptoms or exposures in order to participate. In stage 3, individuals who completed the survey in stage 1 and with self-reported COVID-19 symptoms and positive SARS-CoV-2 IgG antibodies (n = 2313) were sent a follow-up survey between April 9 and May 10, 2021. The follow-up survey was developed to capture the occurrence, duration and most common chronic COVID-19 symptoms. Respondents were asked “Do you still have any lingering symptoms of COVID-19 more than a month after diagnosis?” If they answered yes, they were asked “Which symptoms do you still have? cough, diarrhea, headaches, fatigue/tiredness, loss of smell, change in smell, loss of taste, change in taste, muscle or joint aches, nausea, shortness of breath, stomach pains, weakness, change in hearing, dizziness, other (specify). They were then asked “How long have you now had these lingering symptoms (in months)?” Surveys were administered via the HIPPA-compliant and secure Research Data Capture (REDCap) software. The study was approved by IntegReview institutional review board (Protocol ID: CAPS-613).

### Antibody measurement

Anti-SARS-CoV-2 antibody measurements were performed at the Mayo Clinic Laboratory (Rochester, MN) using the Epitope Diagnostics (EDI) ELISA (San Diego, CA), established and used for clinical reporting of qualitative test for detection of IgM or IgG antibodies to the nucleocapsid protein from SARS-CoV-2. For the purposes of this study, an index value threshold of ≥1.21 was considered positive for COVID-19 [8].

### Data analysis

Baseline characteristics were determined and summary statistics were estimated for individuals who completed the follow-up survey. Frequency and proportion and total number of chronic COVID-19 symptoms were determined overall and stratified by demographics and anti-SARS-CoV-2 antibody levels. Bivariable logistic regression models were constructed to determine whether demographic or household characteristics are associated with having at least one or individual chronic COVID-19 symptoms (dependent variables). Crude odds ratio (OR) and 95% confidence intervals (CI) were estimated. Multivariable models included all variables tested in bivariable models, as well as state of residence as a potential confounder based on theoretical differences in exposures and mitigation strategies. Adjusted OR and 95% CI were estimated. Two- and three-way statistical interactions were tested between covariables.

Medians (interquartile ranges) were estimated for duration of chronic COVID-19 symptoms overall and stratified by characteristics of their acute COVID-19 infection. Mann-Whitney U tests examined differences of chronic COVID-19 duration by characteristics of their acute infection.

Since multiple symptoms can simultaneously occur during acute COVID-19 infection and chronically after COVID-19 infection, latent class analysis (LCA) was used to examine phenotypical patterns of acute COVID-19 symptoms and their impact on occurrence of chronic COVID-19 symptoms, as well as patterns of chronic COVID-19 symptoms. LCA uses observed categorical or binary data to identify patterns, or latent classes. Conditional probabilities were estimated using maximum likelihood to characterize the latent classes by indicating the chance that a member would give a certain response (yes/no) for the specific symptom. Conditional probability plots are presented, where probabilities closer to 0 or 1 indicate lower or higher chances, respectively. LCA regression models examine the differential effects of individual variables across unobserved classes. The ideal number of latent classes and best fitting models were selected by minimizing the Akaike’s information criterion (CAIC) and Bayesian information criterion (BIC) and interpretability. Chi-square tests were used to test the associations of age, sex, household size (above or below the median household size) and the presence of sick contacts in the household with membership in the latent classes.

All data processing and statistical analyses were performed in SAS version 9.4.3 (SAS Institute, Cary, NC, USA). Complete data analysis was performed, i.e. subjects with missing data were excluded. A two-sided p-value <0.05 was considered statistically significant.

## Results

### Population characteristics

Among 6,665 participants who completed the initial phases of the study, 2313 (34.7%) had positive SARS-CoV-2 IgG antibodies. Of these 2313 participants, 390 initiated and 372 completed the follow-up survey (16.1%). The cohort had a median (25%ile, 75%ile) age of 42 (31, 54) years, with 178 females (47.9%) and 356 (95.7%) reporting a history of acute COVID-19 between March-April 2020 (Table 1). The population characteristics were similar to the original cohort characteristics.

**Table 1:** Population characteristics.

Variable	Value	
	Follow-up cohort	Original cohort
N =	372	6,665
Age- median (25%ile, 75%ile)	42 (31, 54)	39.7 (28, 49)
Age- Min–max	18–76	18–94
Female sex–freq (%)	178 (47.9%)	3,068 (46.0%)
Race–freq (%)	347 (93.3%)	NA
Hispanic ethnicity–freq (%)	1 (0.3%)	NA
State of residence–freq (%)		
California	26 (7.0%)	684 (10.3%)
Connecticut	6 (1.6%)	120 (1.8%)
Michigan	9 (2.4%)	339 (5.1%)
New Jersey	188 (50.5%)	3,313 (49.7%)
New York	143 (38.4%)	2,196 (33.0%)
Household size–median (25%ile, 75%ile)	5 (2, 7)	5 (3, 7)
Household size–min, max	1, 15	1, 15
Household sick contact–freq (%)	277 (85.0%)	3,636 (61.4%)
Household sick contact–median (25%ile, 75%ile)	3 (1, 4)	2 (1, 3)
Any symptoms–freq (%)	356 (95.7%)	4,112 (61.7%)
Fever–freq (%)	232 (62.4%)	1,886 (45.9%)
Peak temperature (deg F)–mean ± std. dev.	101.3 ± 1.1	101.0 ± 1.2
Peak temperature (deg F)–min–max	99.0–105.0	99–106
Duration of fever (days)–mean ± std. dev.	3.0 ± 3.3	1.7 ± 2.6
Duration of fever (days)–min–max	<1–10	<1–10
Cough–freq (%)	240 (64.5%)	2,319 (56.6%)
Muscle ache–freq (%)	265 (71.2%)	2,722 (66.4%)
Anosmia–freq (%)	216 (58.1%)	1,966 (48.0%)
Dysgeusia–freq (%)	211 (56.7%)	1,829 (44.6%)
Headache–freq (%)	242 (65.1%)	2,540 (62.0%)
Diarrhea–freq (%)	129 (34.7%)	1,140 (27.8%)
Vomiting–freq (%)	22 (5.9%)	189 (4.6%)
Stomach ache–freq (%)	67 (18.1%)	735 (17.9%)
Required oxygen therapy–freq (%)	8 (2.2%)	36 (0.5%)
Anti-SARS-CoV-2 antibody levels–median (25%ile, 75%ile)	2.2 (1.5, 2.9)	1.0 (1.0, 1.4)

Missing data were encountered in 4 (%) for cigarette smoking.

There were no missing data were encountered for age, sex, household size, household sick contacts, presence of symptoms, any fever or peak fever, duration of fever, requirement of oxygen therapy and anti-SARS-CoV-2 antibody levels.

Similar rates of survey completion were observed across different age groups (97.4% for age 18–49 years, 98.3% for 50–69 years, and 97.8% for 70+ years).

### Prevalence of persistent COVID-19 symptoms

**Fig 2:**
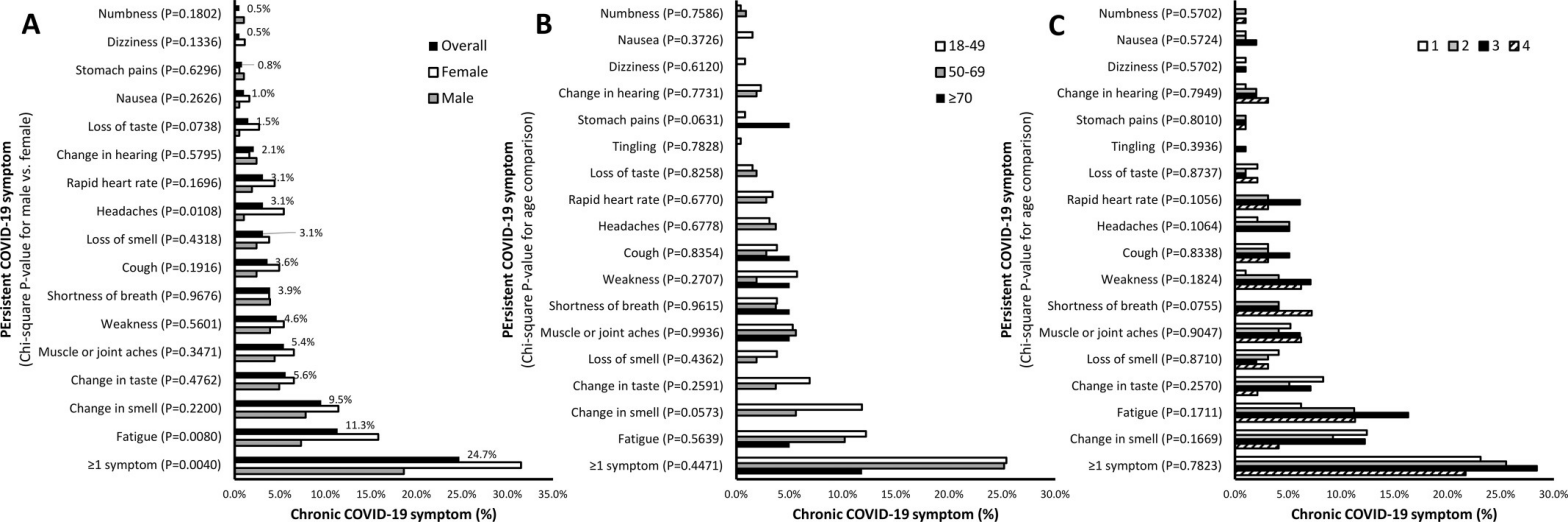
Chronic COVID-19 symptoms. Proportions of chronic COVID-19 symptoms were determined overall (A) and stratified by (A) sex, (B) age and (C) level of SARS-CoV-2 IgG antibody levels. Chi-square tests were performed comparing the frequency of chronic COVID-19 symptoms by sex, age and level of SARS-CoV-2 IgG antibody levels. P-values are presented in the graphs.

Overall, at least one persistent COVID-19 symptom occurred in 92 (24.7%), with 11-month median duration of persistent symptoms (range: 1–12 months). The most common symptom to persist after COVID-19 infection was fatigue (11.3%), followed by change in smell (9.5%) or taste (5.6%), muscle or joint aches (5.4%) and weakness (4.6%) (Fig 2A). Persistent loss of smell (16.5%) or taste (9.3%), muscle or joint ache (7.3%), cough (5.6%) and headache (4.8%) and stomach pain (2.9%) occurred commonly among adults who experienced these symptoms acutely with COVID-19.

There were significantly higher proportions of ≥1 persistent COVID-19 symptom (31.5% vs. 18.6%; Chi-square, P = 0.004), and particularly fatigue (15.8% vs. 7.3%, P = 0.008) and headaches (5.4% vs. 1.0%, P = 0.011) in females compared to males (Fig 2A). There were no significant differences of persistent COVID-19 symptoms by age; though, persistent changes in smell or taste, and fatigue were more prevalent in adults age 18–49 years (Fig 2B). There were no significant differences of persistent COVID-19 symptoms by anti-SARS-CoV-2 antibody levels; though, there was numerically higher prevalence of persistent shortness of breath in adults with highest antibody levels (Fig 2C). There were also no significant differences of persistent COVID-19 symptoms by state of residence (P = 0.140).

### Predictors of persistent COVID-19 symptoms

#### Individual characteristics

Female sex was associated with persistence of ≥1 COVID-19 symptom overall (bivariable logistic regression; OR [95% CI]: 2.02 [1.25–3.26]) (Table 2) and persistent fatigue in particular (2.38 [1.23–4.60]) (Table 3). These associations remained significant in multivariable models.

**Table 2:** Associations of having one or more chronic COVID-19 symptoms.

Variable	≥1 chronic COVID-19 symptom					
	No	Yes	Crude OR (95% CI)	P-value	Adjusted OR (95% CI)	P-value
	Freq (%)	Freq (%)				
*Age (yr)*						
18–49	188 (74.6%)	64 (25.4%)	1.00 [ref]	–	1.00 [ref]	–
50–69	77 (74.8%)	26 (25.2%)	0.99 (0.59–1.68)	0.976	0.95 (0.50–1.81)	0.870
≥70	15 (88.2%)	2 (11.8%)	0.39 (0.09–1.76)	0.221	0.69 (0.13–3.55)	0.653
*Sex*						
Male	158 (81.4%)	36 (18.6%)	1.00 [ref]	–	1.00 [ref]	–
Female	122 (68.5%)	56 (31.5%)	**2.02 (1.25–3.26)**	**0.004**	**1.99 (1.09–3.62)**	**0.025**
*Ever smoked cigarette*						
No	234 (74.6%)	76 (24.5%)	1.00 [ref]	–	1.00 [ref]	–
Yes	42 (72.4%)	16 (27.6%)	1.17 (0.62–2.21)	0.620	1.99 (0.92–4.31)	0.0782
*Household size*						
1–5	131 (78.9%)	35 (21.1%)	1.00 [ref]	–	1.00 [ref]	–
≥6	149 (72.3%)	57 (27.7%)	0.70 (0.43–1.13)	0.144	1.27 (0.72–2.26)	0.414
*Household sick contact*						
No	42 (85.7%)	7 (14.3%)	1.00 [ref]	–	1.00 [ref]	–
Yes	200 (72.2%)	77 (27.8%)	2.31 (0.99–5.36)	0.051	2.25 (0.92–5.49)	0.076

Chronic COVID-19 symptoms assessed included cough, diarrhea, headaches, fatigue/tiredness, loss of smell, change in smell, loss of taste, change in taste, muscle or joint aches, nausea, shortness of breath, stomach pains, weakness, change in hearing, dizziness, and other.

Bivariable logistic regression models were constructed with chronic COVID-19 symptoms (≥1 vs. 0) as the dependent variable and age, sex, household size, household sick contacts as the independent variables. Analyses were limited to persons with positive SARS-CoV-2 anti-IgG antibodies. Crude odds ratios (OR) and 95% confidence intervals (CI) were estimated. Multivariable regression model-1 included all variables from the bivariable models, and state of residence. Adjusted OR and 95% CI were estimated.

Bold indicates statistical significance (P<0.05).

**Table 3:** Associations of chronic fatigue after COVID-19.

Variable	Chronic fatigue					
	No	Yes	Crude OR (95% CI)	P-value	Adjusted OR (95% CI)	P-value
	Freq (%)	Freq (%)				
*Age (yr)*						
18–49	230 (87.8%)	32 (12.2%)	1.00 [ref]	–	1.00 [ref]	–
50–69	97 (89.8%)	11 (10.2%)	0.82 (0.40–1.68)	0.580	0.84 (0.34–2.06)	0.703
≥70	19 (95.0%)	1 (5.0%)	0.38 (0.05–2.92)	0.352	0.97 (0.11–8.56)	0.978
*Sex*						
Male	191 (92.7%)	15 (7.3%)	1.00 [ref]	–	1.00 [ref]	–
Female	155 (84.2%)	29 (15.8%)	**2.38 (1.23–4.60)**	**0.010**	**2.45 (1.05–5.69)**	**0.037**
*Ever smoked cigarette*						
No	280 (88.3%)	37 (11.7%)	1.00 [ref]	–	1.00 [ref]	–
Yes	52 (88.1%)	7 (11.9%)	1.02 (0.43–2.41)	0.966	2.15 (0.75–6.19)	0.156
*Household size*						
1–5	161 (91.5%)	15 (8.5%)	1.00 [ref]	–	1.00 [ref]	–
≥6	184 (86.4%)	29 (13.6%)	1.69 (0.88–3.27)	0.118	1.77 (0.78–4.01)	0.170
*Household sick contact*						
No	49 (94.2%)	3 (5.8%)	1.00 [ref]	–	1.00 [ref]	–
Yes	251 (87.5%)	36 (12.5%)	2.34 (0.69–7.91)	0.170	1.74 (0.50–6.07)	0.388

Bivariable logistic regression models were constructed with fatigue (yes vs. no) as the dependent variable and age, sex, household size, and household sick contacts as the independent variables. Crude odds ratios (OR) and 95% confidence intervals (CI) were estimated. Multivariable regression model-1 included all variables from the bivariable models. Adjusted OR and 95% CI were estimated. Bold indicates statistical significance (P<0.05).

#### Number of acute COVID-19 symptoms

Moreover, a higher number of acute COVID-19 symptoms (3–5, ≥6) was associated with ≥1 chronic COVID-19 symptom overall and fatigue in bivariable and multivariable models, but not chronic anosmia or dysgeusia (Table 4).

**Table 4:** Associations of the number and pattern (latent-class analysis) of acute COVID-19 symptoms with any chronic COVID-19 symptom, particularly chronic fatigue, anosmia and dysgeusia.

**Variable**	**≥1 chronic COVID-19 symptom**					
	**No**	**Yes**	**Crude OR (95% CI)**	**P-value**	**Adjusted OR (95% CI)**	**P-value**
	**Freq (%)**	**Freq (%)**				
**Number of acute COVID-19 symptoms**						
0–2	56 (90.3%)	6 (9.7%)	1.000 (ref)	–	1.000 (ref)	–
3–5	157 (74.4%)	54 (25.6%)	**3.21 (1.31–7.87)**	**0.011**	**3.22 (1.31–7.95)**	0.011
≥6	67 (67.7%)	32 (32.3%)	**4.46 (1.74–11.42)**	**0.002**	**4.23 (1.64–10.93)**	**0.003**
**Pattern of acute COVID-19 symptoms (latent-class analysis)**						
1. Highest probability of all symptoms	32 (64.0%)	18 (36.0%)	**3.70 (1.38–9.88)**	**0.009**	**3.62 (1.33–9.83)**	**0.012**
2. Lowest probability of all symptoms	46 (86.8%)	7 (13.2%)	1.000 (ref)	–	1.00 (ref)	–
3. Fever, cough, muscle ache, anosmia, dysgeusia, headache	135 (72.6%)	51 (27.4%)	**2.48 (1.05–5.85)**	**0.038**	2.32 (0.97–5.55)	0.059
4. Fever, cough, muscle ache, headache	68 (80.0%)	17 (20.0%)	1.64 (0.63–4.28)	0.309	1.63 (0.65–4.02)	0.322
	**Chronic fatigue**					
	**No**	**Yes**	**Crude OR (95% CI)**	**P-value**	**Adjusted OR (95% CI)**	**P-value**
	**Freq (%)**	**Freq (%)**				
**Number of acute COVID-19 symptoms**						
0–2	67 (98.5%)	1 (1.5%)	1.00 (ref)	–	1.00 (ref)	–
3–5	196 (89.1%)	24 (10.9%)	**8.20 (1.09–61.82)**	**0.041**	**8.48 (1.12–64.17)**	**0.038**
≥6	82 (81.2%)	19 (18.8%)	**15.52 (2.03–118.99)**	**0.008**	**14.62 (1.90–112.56)**	**0.01**
**Pattern of acute COVID-19 symptoms (latent-class analysis)**						
1. Highest probability of all symptoms	40 (78.4%)	11 (21.6%)	**7.84 (1.65–37.29)**	**0.01**	**7.36 (1.53–35.47)**	**0.013**
2. Lowest probability of all symptoms	57 (96.6%)	2 (3.4%)	1.00 (ref)	–	1.00 (ref)	–
3. Fever, cough, muscle ache, anosmia, dysgeusia, headache	170 (88.1%)	23 (11.9%)	3.86 (0.88–16.87)	0.073	3.58 (0.81–15.85)	0.094
4. Fever, cough, muscle ache, headache	80 (90.9%)	8 (9.1%)	2.85 (0.58–13.92)	0.196	2.84 (0.58–13.96)	0.2
	Chronic anosmia					
	No	Yes	Crude OR	P-value	Adjusted OR	P-value
	Freq (%)	Freq (%)	(95% CI)		(95% CI)	
**Number of acute COVID-19 symptoms**						
0–2	65 (95.6%)	3 (4.4%)	1.00 (ref)	–	1.000 (ref)	–
3–5	195 (88.6%)	25 (11.4%)	2.78 (0.81–9.50)	0.226	2.81 (0.82–9.70)	0.102
≥6	92 (91.1%)	9 (8.9%)	2.12 (0.55–8.13)	0.247	2.01 (0.52–7.77)	0.313
**Pattern of acute COVID-19 symptoms (latent-class analysis)**						
1. Highest probability of all symptoms	46 (90.2%)	5 (9.8%)	1.17 (0.32–4.31)	0.809	1.00 (0.27–3.71)	0.997
2. Lowest probability of all symptoms	54 (91.5%)	5 (8.5%)	1.00 (ref)	–	1.00 (ref)	–
3. Fever, cough, muscle ache, anosmia, dysgeusia, headache	167 (86.5%)	26 (13.5%)	1.68 (0.61–4.59)	0.311	1.47 (0.53–4.08)	0.462
4. Fever, cough, muscle ache, headache	86 (97.7%)	2 (2.3%)	0.25 (0.05–1.34)	0.106	0.24 (0.04–1.27)	0.092
	**Chronic dysgeusia**					
	**No**	**Yes**	**Crude OR**	**P-value**	**Adjusted OR**	**P-value**
	**Freq (%)**	**Freq (%)**	**(95% CI)**		**(95% CI)**	
**Number of acute COVID-19 symptoms**						
0–2	67 (98.5%)	1 (1.5%)	1.00 (ref)	–	1.00 (ref)	–
3–5	202 (91.8%)	18 (8.2%)	5.97 (0.78–45.57)	0.085	6.03 (0.79–46.31)	0.084
≥6	98 (97.0%)	3 (3.0%)	2.05 (0.21–20.14)	0.538	1.93 (0.20–19.04)	0.574
**Pattern of acute COVID-19 symptoms (latent-class analysis)**						
1. Highest probability of all symptoms	48 (94.1%)	3 (5.9%)	1.17 (0.23–6.05)	0.854	0.97 (0.19–5.12)	0.975
2. Lowest probability of all symptoms	56 (94.9%)	3 (5.1%)	1.00 (ref)	–	1.00 (ref)	–
3. Fever, cough, muscle ache, anosmia, dysgeusia, headache	177 (91.7%)	16 (8.3%)	1.69 (0.47–6.00)	0.419	1.49 (0.41–5.40)	0.545
4. Fever, cough, muscle ache, headache	87 (98.9%)	1 (1.1%)	0.22 (0.02–2.12)	0.187	0.20 (0.02–1.95)	0.165

Bivariable logistic regression models were constructed with ≥1 chronic COVID-19 symptom or chronic fatigue, anosmia and dysgeusia (yes vs. no) as the dependent variable and number of acute COVID-19 symptoms (0-2/3-5/≥6) and pattern of acute COVID-19 symptoms (ordinal variable with 4-classes derived from latent-class analysis) as the independent variable. Analyses were limited to persons with positive SARS-CoV-2 anti-IgG antibodies. Crude odds ratios (OR) and 95% confidence intervals (CI) were estimated. Multivariable models included age (continuous), sex (male/female), race (white/non-white) and Hispanic ethnicity (yes/no) as covariables, and state of residence. Adjusted OR and 95% CI were estimated.

Bold-face indicates statistical significance (P<0.05).

#### Pattern of acute COVID-19 symptoms

We used LCA to identify patterns of acute COVID-19 symptoms. The best-fit model had four classes. Class-1 had the lowest membership probability (13.1% of the cohort) and consisted of high probabilities of all symptoms. Class-2 had the next highest membership probability (14.9% of the cohort) and had lowest probabilities of all symptoms. Class-3 (49.4% of the cohort) had highest probability of membership and high probabilities of fever, cough, muscle ache, anosmia, dysgeusia, and headache. Class-4 (22.6% of the cohort) had higher probabilities of fever, cough, muscle ache, and headache. Membership in class-1 was associated with significantly higher odds of ≥1 chronic COVID-19 symptom and fatigue in bivariable and multivariable regression models, but not chronic anosmia or dysgeusia (Table 3).

There were no significant two- or three-way statistical interactions in the abovementioned models.

#### Anti-SARS-CoV-2 antibodies

Moreover, levels of anti-SARS-CoV-2 antibodies were not consistently associated with ≥1 chronic COVID-19 symptom overall, fatigue, anosmia or dysgeusia in bivariable or multivariable models (Table 5).

**Table 5:** Associations of anti-SARS-CoV-2 antibody levels with any chronic COVID-19 symptom, particularly chronic fatigue, anosmia and dysgeusia.

Anti-SARS-CoV-2 antibody level–quartile	≥1 chronic COVID-19 symptom					
	No	Yes	Crude OR (95% CI)	P-value	Adjusted OR (95% CI)	P-value
	Freq (%)	Freq (%)				
1	70 (76.9%)	21 (23.1%)	1.00 (ref)	–	1.00 (ref)	–
2	70 (74.5%)	24 (25.5%)	1.14 (0.58–2.24)	0.697	1.24 (0.62–2.47)	0.547
3	68 (71.6%)	27 (28.4%)	1.32 (0.68–2.56)	0.406	1.34 (0.67–2.71)	0.411
4	72 (78.3%)	20 (21.7%)	0.93 (0.46–1.86)	0.828	1.02 (0.48–2.15)	0.961
	**Chronic fatigue**					
	**No**	**Yes**	**Crude OR (95% CI)**	**P-value**	**Adjusted OR (95% CI)**	**P-value**
	**Freq (%)**	**Freq (%)**				
1	91 (93.8%)	6 (6.2%)	1.00 (ref)	–	1.00 (ref)	–
2	86 (88.7%)	11 (11.3%)	1.94 (0.69–5.47)	0.211	2.27 (0.79–6.53)	0.129
3	82 (83.7%)	16 (16.3%)	**2.96 (1.11–7.92)**	**0.031**	**3.26 (1.17–9.05)**	**0.024**
4	86 (88.7%)	11 (11.3%)	1.94 (0.69–5.47)	0.211	2.53 (0.84–7.60)	0.098
	**Chronic anosmia**					
	**No**	**Yes**	**Crude OR (95% CI)**	**P-value**	**Adjusted OR (95% CI)**	**P-value**
	**Freq (%)**	**Freq (%)**				
1	85 (87.6%)	12 (12.4%)	1.00 (ref)	–	1.00 (ref)	–
2	88 (90.7%)	9 (9.3%)	0.72 (0.29–1.81)	0.490	0.83 (0.33–2.12)	0.702
3	86 (87.8%)	12 (12.2%)	0.99 (0.42–2.32)	0.979	1.18 (0.48–2.90)	0.717
4	93 (95.9%)	4 (4.1%)	**0.31 (0.10–0.98)**	**0.046**	0.41 (0.12–1.39)	0.151
	**Chronic dysgeusia**					
	**No**	**Yes**	**Crude OR (95% CI)**	**P-value**	**Adjusted OR (95% CI)**	**P-value**
	**Freq (%)**	**Freq (%)**				
1	89 (91.8%)	8 (8.3%)	1.00 (ref)	–	1.00 (ref)	–
2	92 (94.9%)	5 (5.2%)	0.61 (0.19–1.92)	0.393	0.68 (0.21–2.20)	0.520
3	91 (92.9%)	7 (7.1%)	0.86 (0.30–2.46)	0.772	0.99 (0.33–2.99)	0.984
4	95 (97.9%)	2 (2.1%)	0.23 (0.05–1.13)	0.071	0.30 (0.06–1.53)	0.146

Anti-SARS-CoV-2 antibody level quartile-1: 1.02–1.46; quartile-2: 1.47–2.14; quartile-3: 2.15–2.91; quartile-4: 2.92–4.40

Bivariable logistic regression models were constructed with ≥1 chronic COVID-19 symptom or chronic fatigue, anosmia or dysgeusia (yes vs. no) as the dependent variable and level of anti-SARS-CoV-2 antibodies (quartiles) as the independent variable. Analyses were limited to persons with positive SARS-CoV-2 anti-IgG antibodies. Crude odds ratios (OR) and 95% confidence intervals (CI) were estimated. Multivariable models included age (continuous), sex (male/female), race (white/non-white) and Hispanic ethnicity (yes/no) as covariables, and state of residence. Adjusted OR and 95% CI were estimated.

Bold-face indicates statistical significance (P<0.05).

### Duration of persistent COVID-19 symptoms

**Fig 3:**
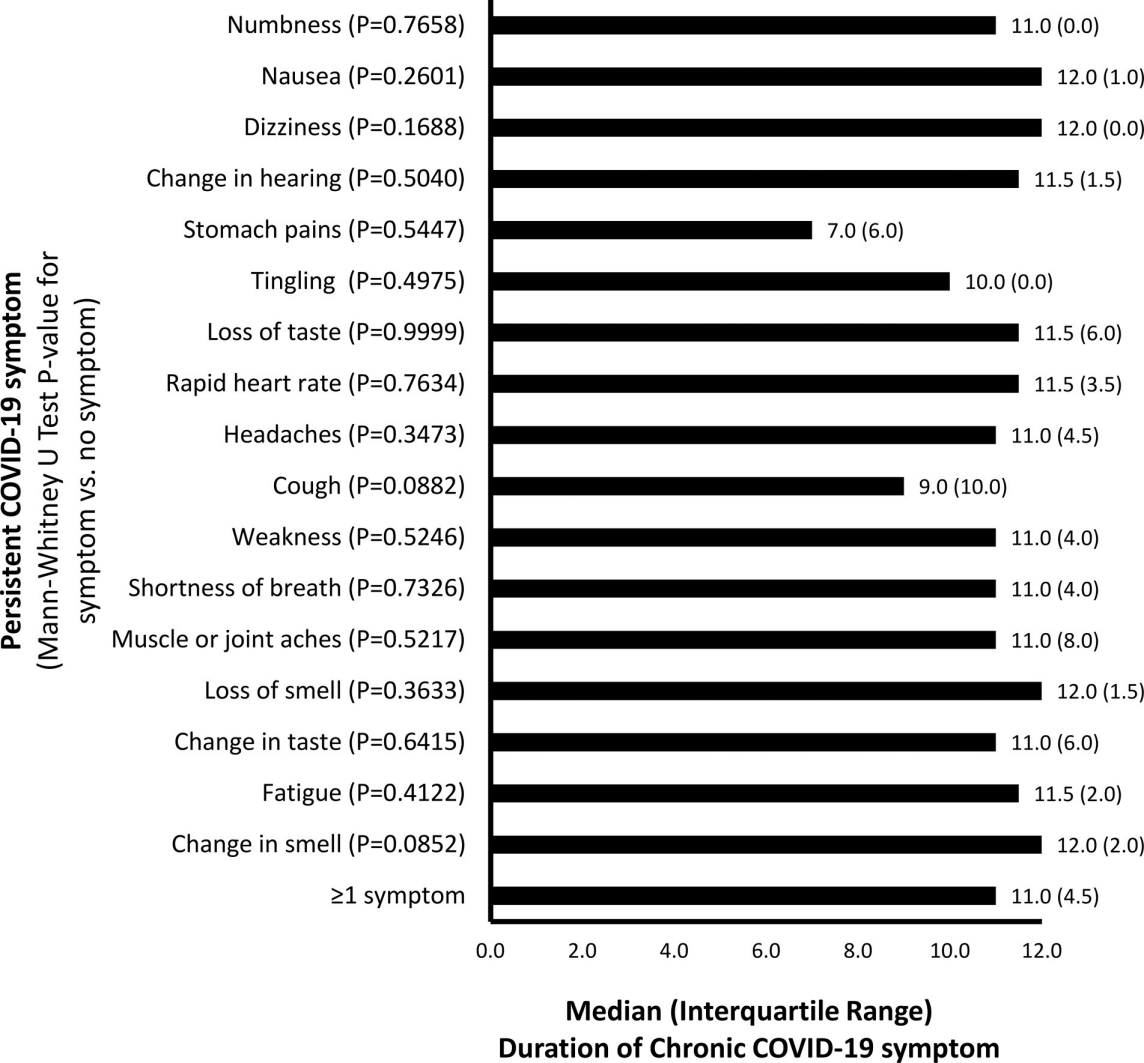
Duration of chronic COVID-19 symptoms. Median duration of chronic COVID-19 symptoms were determined overall and stratified by individual symptoms. Mann-Whitney U tests were performed comparing the duration of chronic COVID-19 symptoms between those with vs. without a history of acute COVID-19 symptoms. P-values are presented in the graphs.

Overall, the median (25%ile, 75%ile) duration of persistent COVID-19 symptoms was 11 (8, 12) months. Median duration of persistence was similar for most symptoms (11–12 months), but numerically shorter for stomach pains (7 months), cough (9 months) and tingling (10 months) (Mann-Whitney U test, P≥0.08)(Fig 3). Median [25%ile, 75%ile] duration of persistent COVID-19 symptoms was significantly higher in individuals with higher fever (<102 degF: 9 [6, 12]; 102–106 deg F: 12 [11, 12]; P = 0.041), prolonged fever (<7 days: 11 [6, 12]; ≥7 days: 12 [11, 12]; P = 0.026) and numerically higher among those with more COVID-19 symptoms (<3 symptoms: 8 [3, 11]; ≥3 symptoms: 11 [9, 12]; P = 0.099) during acute infection.

### Patterns of chronic COVID-19 symptoms

**Fig 4:**
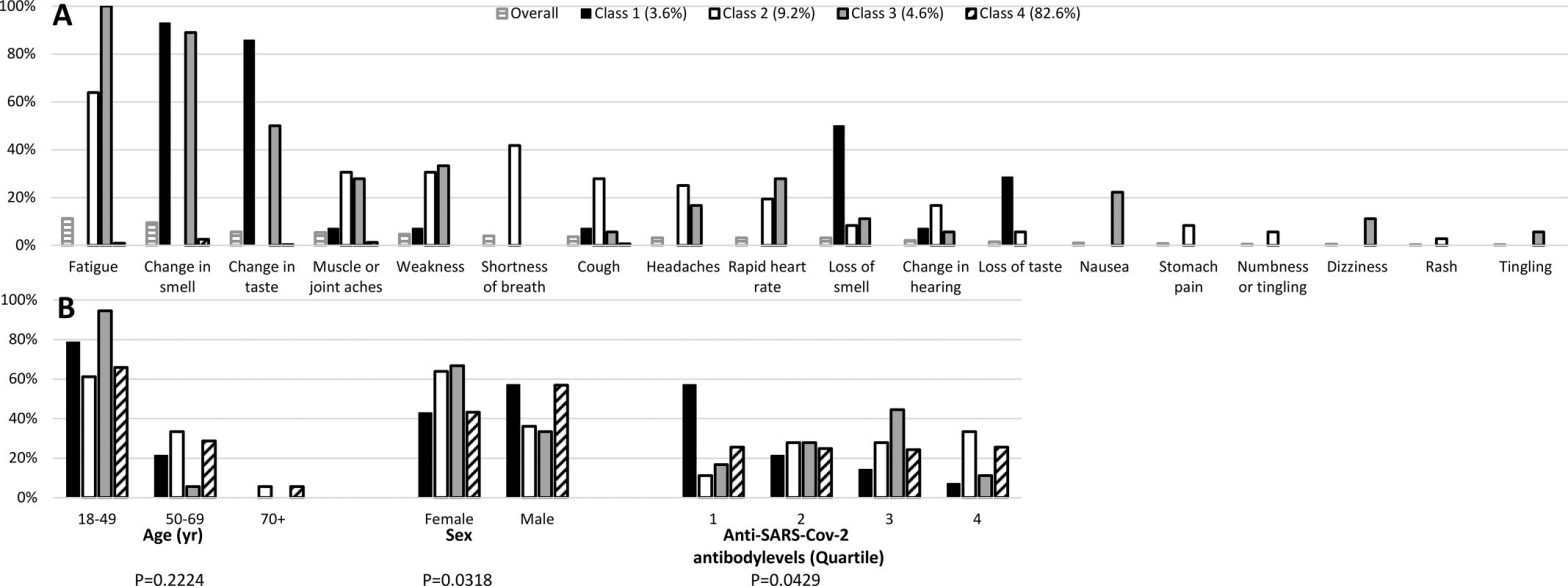
Latent class analysis of patterns of chronic COVID-19 symptoms. Latent Class Analysis was used to examine patterns of binary variables of chronic SARS-CoV-2 symptoms in adults. LCA used the observed binary data to identify homogeneous patterns, i.e. n = 4 latent classes. Conditional probabilities were estimated using maximum likelihood to characterize the latent classes. (A) Conditional probability plots are presented, where probabilities closer to 0 or 1 indicate lower or higher chances, respectively. The horizontal black lines represent the overall distribution of SARS-CoV-2 symptoms. The black solid fill represents Class 1. The white solid fill represents Class 2. The gray solid fill represents Class 3. The diagonal black lines represent Class 4. The proportion of respondents who are members of these classes is presented. B. Chi-square tests were performed comparing (B) age, sex and SARS-CoV-2 IgG antibody levels with class membership.

We also used LCA to identify statistically significant homogeneous patterns of chronic COVID-19 symptoms. The best-fit model had four classes. Conditional probabilities of having persistent COVID-19 symptoms are plotted in Fig 4A. Class-4 had the highest membership probability (82.6% of the survey cohort) and consisted of very low probabilities of any chronic COVID-19 symptoms. Class-2 had the next highest membership probability (9.2% of the survey cohort) and had higher probabilities of chronic fatigue, muscle aches, weakness, shortness of breath, cough, headaches, rapid heart rate, change in hearing, and stomach pain. Class-3 (4.6% of the survey cohort) had higher probabilities of chronic fatigue, change in smell and taste, muscle aches, weakness, nausea, and dizziness. Class-1 (3.6% of the survey cohort) had higher probabilities of chronic changes of smell and taste.

There were significant associations of latent class membership with sex (Chi-square, P = 0.032), and level of anti-SARS-CoV-2 antibodies (Fisher exact test, P = 0.0429), but not age (P = 0.222) (Fig 4B). Membership in Classes-2 and 3 (higher probabilities of chronic COVID-19 symptoms) was higher in females. Class-4 membership (no chronic symptoms) was evenly distributed across anti-SARS-CoV-2 antibody levels. Whereas, Class-1 membership was associated with lower anti-SARS-CoV-2 antibody levels, and Class-2 and 3 membership was associated with higher anti-SARS-CoV-2 antibody levels.

## Discussion

In this longitudinal observational community-based cohort of adults who were not previously vaccinated for COVID-19 and had confirmed COVID-19 infection, approximately 1 in 4 reported at least one persistent COVID-19 symptom, and 1 in 10 reported chronic fatigue and change in smell. The most common persistent symptoms were fatigue, change in smell or taste, muscle/joint aches and/or weakness. Persistent COVID-19 symptoms overall and chronic fatigue in particular were more common in females and individuals with higher number and more severe acute COVID-19 symptoms. Previous studies found fatigue to be the most frequent chronic COVID-19 symptom, with conflicting results regarding association of acute COVID-19 symptoms with chronic fatigue [2, 9–11]. A previous Italian study of 223 patients who were infected by SARS-CoV-2 similarly found that females were more likely to experience persistent COVID-19 symptoms, including dyspnea, fatigue, chest pain, and palpitations [12]. Interestingly, we found that older age was not associated with having ≥1 or patterns of chronic COVID-19 symptoms. Additionally, duration of persistent COVID-19 symptoms was prolonged among individuals with high fever, prolonged fever and more COVID-19 symptoms during acute infection. Together, the results suggest that severity of acute COVID-19 infection is the strongest predictor of chronic COVID-19 symptoms. Of note, chronic anosmia and dysgeusia were not consistently associated with more severe acute COVID-19 or antibody levels, suggesting different factors contribute to these symptoms.

A previous Irish study of 223 persons with confirmed diagnosis of COVID-19 and persistent symptoms >4 weeks after onset of acute symptoms identified three different symptom clusters, including prominent pain-related symptoms, cardiopulmonary symptoms, and fewer symptoms. Individuals with pain-related or cardiopulmonary symptoms had greater functional impairment, work absence, higher dyspnea scores, and poorer health-related quality of life [13]. Our study also identified a cluster of patients with increased cardiopulmonary symptoms, but also found clusters with persistent changes of smell and taste in isolation, changes of smell and taste in combination with chronic fatigue and other somatic symptoms.

Previous studies showed complications of COVID-19 affecting virtually every organ system [14]. This study helps answer several questions previously identified as important unmet research needs: How many individuals will suffer from chronic COVID-19 symptoms and which symptoms they may suffer from; the duration of chronic symptoms; and features of acute COVID-19 which predict chronic COVID-19 symptoms [3]. Our study is consistent with a prior prospective analysis that revealed a prevalence of chronic COVID-19 symptoms of 30% at 6 months, with our study additionally noting persistence to a median 11 months [1]. Interestingly, a recent meta-analysis by Groff et. al. showed an even higher incidence of chronic COVID-19 symptoms (54% at 6 months) [2].

Higher anti-SARS-CoV-2 IgG levels were not associated with having ≥1 chronic COVID-19 symptom overall but were associated with specific patterns of chronic COVID-19 symptoms. Specifically, higher antibody levels were associated with more severe patterns of chronic COVID-19 symptoms, whereas isolated chronic changes of smell and taste were associated with lower antibody levels. These findings are most likely explained by higher anti-SARS-CoV-2 IgG levels occurring in more severe acute COVID-19 infection. Our results also suggest that mounting a strong humoral immune response does not protect against developing chronic COVID-19 symptoms. This is consistent with previous studies that found SARS-CoV-2 replicates intracellularly within a self-limited period of time and does not generally cause chronic cellular infection; a robust immune response leads to viral clearance [15, 16]. Thus, most chronic COVID-19 symptoms are likely attributable to other mechanisms, including viral toxicity and hyper-immune response leading to persistent end-organ damage, e.g. lung damage leading to chronic cough or dyspnea or encephalopathy leading to chronic fatigue and neurologic symptoms [17]. Alternatively, secondary immune activation may result in production of auto-antibodies that target different organs long after the virus is cleared, e.g. perinuclear anti-neutrophil cytoplasmic antibodies, rheumatoid factor, antibodies against prothrombin and different nervous system antigens [18–21]. In any case, identification of a correlation between chronic COVID-19 symptoms with seropositivity may beget the study of novel pathophysiologic mechanisms of chronic COVID-19 and of postacute illness more generally.

There were several intriguing findings regarding temporal patterns of chronic COVID-19 symptoms. Specific symptoms, e.g. anosmia and muscle ache, were more likely to occur chronically among those who initially reported having those symptoms during acute COVID-19 infection. On the other hand, many individuals who endorsed specific chronic COVID-19 symptoms in the follow-up survey did not initially report having those symptoms acutely in the baseline survey. This suggests that some of the chronic symptoms may have had a delayed onset. However, the total frequency of delayed onset chronic symptoms was not high enough to allow a more detailed analysis of its predictors. Of note, this study included a community-based cohort sampled between May and July, 2020 during the initial wave of COVID-19 infections when vaccines were unavailable. The results may not be generalizable to other SARS-CoV-2 variants. Future studies are needed to better understand COVID-19 variants, as well as delayed-onset chronic COVID-19 symptoms and how these differ from persistent acute-onset symptoms.

This study included of a wide age-range and spectrum of disease severity, including a fairly high proportion of younger adults and milder symptoms. This allowed for comparison of chronic COVID-19 symptoms as a function of age and acute COVID-19 severity. However, there are limitations. This ambulatory cohort may not accurately reflect the chronic COVID-19 symptom profile of patients with very severe acute disease. We confirmed COVID-19 infection using antibodies to the nucleocapsid protein, which may have a different symptom profile than those with positive antibodies to the spike protein. This was a largely Ashkenazi Jewish population. COVID-19 hit the Orthodox Jewish community in the United States particularly hard, especially in the early days when much was unknown. At that time of great loss, Jewish communities around the United States rallied to participate in research to help the millions of other people impacted by the pandemic. While the cohort included broad representation of age and sex, there was limited racial diversity. The low response rate for follow-up surveys may be due to non-response bias, as individuals who do not experience symptoms of chronic COVID-19 may have been less likely to complete the follow-up survey. This may lead to overestimates of chronic COVID-19 symptoms. Further, the smaller sample size may lead to lower precision of estimates and potential Type II error. There are additional potential confounders that were not assessed, including socioeconomic status and presence of major comorbidities associated with greater COVID-19 severity, including obesity, diabetes, dyslipidemia, and possibly geography (urban vs. rural). Future studies are needed to address these limitations.

In conclusion, chronic COVID-19 symptoms commonly occur in previously unvaccinated adults who experience COVID-19 infection and can persist for many months, especially fatigue, changes in smell or taste and muscle/joint aches. Adults who were female, experienced more severe acute COVID-19 infection or who had higher anti-SARS-CoV-2 IgG levels were at highest risk of having chronic COVID-19 symptoms overall and fatigue in particular. Future studies should examine the specific mechanisms of different chronic COVID-19 symptoms and optimal strategies to prevent and treat chronic COVID-19 symptoms.

## Abbreviations

BIC: Bayesian information criterion
CAIC: corrected Akaike information criterion
CI: confidence interval
IgG: Immunoglobulin G
LCA: latent class analysis
OR: odds ratio
SD: standard deviation
